# Institution-Wide Retreats Foster Organizational Learning and Action at a Comprehensive Cancer Center

**DOI:** 10.1007/s13187-024-02418-9

**Published:** 2024-03-12

**Authors:** Benjamin R. Schrank, John A. Fuller, Colleen M. Gallagher, Van K. Morris, Emma B. Holliday, Kelly Merriman, Lynne Nguyen, Lou Weaver, Kelly Nelson, Elizabeth Chiao, Albert C. Koong, Ernest Hawk, Shine Chang

**Affiliations:** 1 The University of Texas, MD Anderson Cancer Center (Radiation Oncology), 1400 Pressler St, Houston, TX 77030 USA; 2 Nell Hodgson Woodruff School of Nursing, Emory University, Atlanta, GA USA; 3 The University of Texas, MD Anderson Cancer Center (Critical Care Medicine), Houston, TX USA; 4 The University of Texas, MD Anderson Cancer Center (Cancer Medicine), Houston, TX USA; 5 The University of Texas, MD Anderson Cancer Center (Cancer Registry), Houston, TX USA; 6 The University of Texas, MD Anderson Cancer Center (Health Disparities Research), Houston, TX USA; 7 The University of Texas, MD Anderson Cancer Center (Epidemiology), Houston, TX USA; 8 The University of Texas, MD Anderson Cancer Center (Dermatology), Houston, TX USA; 9 The University of Texas, MD Anderson Cancer Center (Clinical Cancer Prevention, Cancer Prevention & Population Science), Houston, TX USA

**Keywords:** Sexual and gender minority, Sexual orientation and gender identity, Data collection, Patient education, Staff training, Institution-wide retreat

## Abstract

Providing safe and informed healthcare for sexual and gender minority (SGM) individuals with cancer is stymied by the lack of sexual orientation and gender identity (SOGI) data reliably available in health records and by insufficient training for staff. Approaches that support institutional learning, especially around sensitive topics, are essential for hospitals seeking to improve practices impacting patient safety and research. We engineered annual institutional retreats to identify and unify stakeholders, promote awareness of gaps and needs, identify initiatives, minimize redundant projects, and coordinate efforts that promote improvements in SGM cancer care, education, and research. The 2022 and 2023 retreats employed a 4-h hybrid format allowing virtual and in-person engagement. Retreat organizers facilitated small-group discussions for brainstorming among participants. We performed descriptive statistics from retreat evaluations. The retreats engaged 104 attendees from distinct departments and roles. Participants expressed robust satisfaction, commending the retreat organization and content quality. Notably, the first retreat yielded leadership endorsement and funding for a Quality Improvement pilot to standardize SOGI data collection and clinical staff training. The second retreat provided a platform for updates on focused efforts across the institution and for receiving direction regarding national best practices for SGM care and research. We report the processes and outcomes of institution-wide retreats, which served as a platform for identifying gaps in organizational healthcare practices and research for SGM individuals with cancer. The strategies described herein may be readily scaled at other cancer hospitals seeking to learn and enact system-wide practice changes that support the needs of SGM patients and families.

## Introduction

Disparities in cancer-related outcomes endure when failures to prioritize patient safety and respect foster institutional inattention to the needs of marginalized people. For sexual and gender minority (SGM) individuals with cancer, the failure by clinical staff to acknowledge their sexual orientation and gender identity (SOGI) status perpetuates discomfort in and avoidance of life-saving medical encounters [1–3]. For oncology staff, the lack of SOGI documentation in health records threatens their provision of safe and informed oncologic care [4]. Most hospitals and hospital systems have not yet organized internal efforts to create and implement such processes despite available fields provided by electronic health record vendors made mandatory by the Affordable Care Act in 2014 [5]. In 2022, the National Cancer Institute (NCI) organized a funding opportunity for administrative supplements for P30 cancer center support grants with an aim to stimulate plans to collect such data within cancer centers.

Large institutions, including cancer centers, must contend with distinct challenges in aligning cultures of patient safety and respect across multiple levels of their organization from institutional leadership to multidisciplinary clinics and specialized research teams. To initiate and implement system-wide changes, institutions use a variety of strategies to engage staff and stakeholders both widely across campuses and in microenvironment settings. For example, workshops and didactic training can focus on building skills for medical team performance, thereby improving clinical processes [6–8]. In contrast, retreats can serve to introduce institutional initiatives in nascent phases of development and promotion [9], and by bringing together staff with diverse roles and responsibilities, they can orient and unify stakeholders toward shared goals. Retreats by communicating information among administrators, policymakers, researchers, patient advocates, educators, and practitioners of varying specialties can also expand the use of best practices and also reduce redundant efforts. Strategically, retreats can promote awareness of initiatives that may be organizationally perceived as sensitive or controversial that must still progress despite individual hesitation or discomfort.

The objective of this paper is to present the organizational strategies, processes, and outcomes of institutional retreats at a large, state university-affiliated NCI-designated comprehensive cancer center held in the setting of a new Texas State law focused on removing diversity, equity, and inclusion (DEI) efforts in state agencies, including universities, [10, 11] which became operative in January 2024.

## Methods

### Committee Formation and Mission

Composed of nine interdisciplinary individuals from various departments, including Epidemiology, Cancer Registry, Clinical Cancer Prevention, Health Disparities Research, Medical Oncology, Radiation Oncology, and Clinical Ethics, the committee was self-organized in 2021 and includes faculty, administrators, researchers, and clinicians. This core group shared a vision to identify and implement culturally appropriate, institutionally sustainable interventions and patient-focused projects to improve cancer care and research for SGM health, not to change individual attitudes but to affect professional behavior relevant to patient care and research. The committee included faculty and staff who played integral roles in prior system-wide practice changes to implement processes to uniformly collect and report gender, race, and ethnicity data among all newly and previously registered patients in 2014 following the installation of a new electronic health record aligned with federal reporting requirements.

The committee was formed to address foundational gaps around institutional SOGI data collection and management. Although patients could self-identify their SOGI information through their electronic health record portal before the committee’s formation, the absence of context or support provided by patients’ clinical teams resulted in sporadic and low rates of completion of SOGI data fields by patients. While physicians and advanced practice providers were institutionally recognized as potential contributors to the accurate collection of SOGI data, the lack of standardized workflows and assigned responsibility for eliciting and recording data also led to low completion rates. Concurrently, limited staff training regarding data collection and appropriate use of SOGI data in patient charts and encounters existed, inhibiting the confident and professional behavior desired of staff.

### Need for Institutional Learning and Engagement

The committee’s proactive stance and institution’s mission of continuously improving patient-directed care led to submission in January 2021 of an administrative supplement to the NCI, outlining a nationwide collaboration to collect SOGI patient data across academic medical centers. The initial success of garnering executive leadership support for the supplement motivated the committee to consider other ways to foster institutional learning and engagement, including inviting speakers or organizing poster sessions on SGM education and research with institutional and external meeting participants. Ultimately, the rationale for organizing an institutional retreat prevailed, grounded in the premise that such an event could provide a platform for institution-wide engagement and open exchange of ideas from diverse stakeholders. The committee recognized that potential action plans resulting from the open forum might conflict with other institutional initiatives, and so worked with leaders to plan the retreat to avoid such conflicts. The committee also managed activities of the retreat to abide by state policies and procedures for the institution, a state university-affiliated healthcare organization.

### Retreat Design

Two half-day institutional retreats have now been held in 2022 and 2023. Organized in 4-h blocks, the agendas had a primary emphasis on fostering engagement and candid dialog through small-group sessions. A virtual option for attendance facilitated participation by staff working remotely due to the COVID-19 pandemic or unable to leave clinical posts; however, participants were strongly encouraged to attend in person. The retreats were held in conference rooms equipped with audiovisual capabilities to facilitate interaction between in-person and virtual attendees and presenters.

Throughout planning, several strategic decisions were made to enhance success. First, although the retreat was open to all 24,000 institutional employees, targeted invitations were used to secure the representation of executive leadership, division chairs, and patient advocates in retreat discussions reflecting a range of institutional perspectives and lived experiences. Second, the retreat began with brief presentations on SGM cancer care, education, and research to prime ideas that could be developed in smaller group discussions into actions and plans. Third, limiting discussion groups to 15 participants helped facilitate robust conversations. Fourth, retreats were scheduled in June to coincide with institutional attention toward Pride-related events, helping foster a supportive environment. Fifth, senior leaders were actively involved in planning to ensure the committee goals aligned with leadership-approved strategies. Finally, interprofessional teams of faculty members, executive leaders, and trainees served as retreat organizers to facilitate more informative and collaborative discussions in small groups.

### Retreat Content

The 2022 retreat commenced with a large-group session including an introduction to the retreat’s objectives and short talks selected by the committee focused on patient education, staff training, and clinical research. Following the presentations, attendees transitioned to small-group sessions facilitated by four interprofessional faculty. These sessions emphasized fostering collaborative discussions among frontline staff, senior executives, physicians, researchers, and ancillary staff on patient-centered care, education, and research for SGM populations.

To develop specific, measurable, achievable, relevant, and time-bound (SMART) initiatives, the small groups conducted discussions focused on two areas:

1. Expanding clinical and research ideas to facilitate the care and study of SGM populations. Facilitators elicited views on the collection of SOGI data and how data may be used to further the study of risks, prevalence, and oncologic outcomes for SGM patients.
2. Identifying critical components needed for creating a safe and welcoming environment for SGM patients and staff, including for staff education for patient safety. Facilitators elicited ideas on training resources and strategies needed for creating a more welcoming and comfortable patient environment in physical and electronic spaces.

Following the small-group sessions, facilitators regrouped to discuss next steps based on the participants’ goals. Activities included reviewing statements made and identifying collaborators for future work. The event concluded with time for participants to critically evaluate the retreat.

### Retreat Content

The 2023 retreat commenced with a large-group session spotlighting progress on institutional initiatives stemming from the 2022 retreat. This session was followed by a keynote address delivered by the Director of the SGM Research Office at the NIH, Dr. Karen Parker focused on best practices in data collection, analysis, research, and reporting. Subsequent small-group discussions focused on advancing ongoing projects and conceiving novel initiatives.

### Logistics/Preparation

Preparation for each years’ retreat was initiated 3 months in advance to secure venue reservations, coordinate schedules, and ensure clinical coverage for participating front-line staff. The committee held regular meetings to review the agenda, logistics, strategies for engaging virtual attendees, and goals. Post-retreat, debriefing sessions were conducted to share insights and pinpoint areas for future enhancement.

### Evaluation

As the purpose of the retreat was not to impart specific knowledge of SOGI terms or healthcare disparities within the LGBTQ community, the retreat’s evaluation scored participant engagement during the retreat and overall satisfaction. The evaluations were collected via structured online forms in 2022 and 2023. These questionnaires featured both quantitative and qualitative components; the 2022 form consisted of seven questions and achieved a 56% (27/46) response rate, and the 2023 form comprised 30 questions and obtained a 36% (21/58) response rate.

### Data Analysis

Descriptive statistics were used to analyze survey items. All responses from open-ended questions were compiled for review by the committee.

## Results

### Leadership Endorsement

The 2022 and 2023 retreats were supported by the institution’s Chief Medical Executive, the Chief Diversity, Equity, and Inclusion Officer, and the Vice President of Cancer Prevention. As a result of the 2022 retreat, the Chair of the Division of Radiation Oncology invited committee members to apply for institutional funding to support initiatives developed during the retreat for patient-oriented cancer care, including a pilot study to test the feasibility of standardizing SOGI data collection and training for both staff responsible for collecting data and clinic staff interacting with patients and their families. Following the 2022 retreat, committee members were invited to share updates on retreat outcomes at various leadership meetings including a meeting of the Division Heads.

### Retreat Participation and Participant Satisfaction

**Fig. 1:**
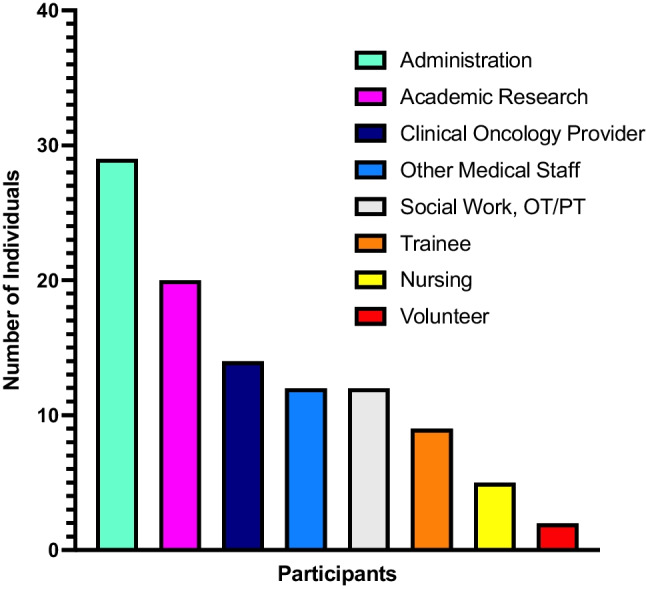
Number of retreat participants in 2022 and 2023 retreats by institutional role

The institutional retreats held in 2022 and 2023 saw active participation from a total of 104 attendees representing various divisions and sectors of the institution (Fig. 1). The attendees came from 22 unique departments and offices (Table 1).

**Table 1:** Departments and offices represented at retreats

Clinical care	Clinical operations	Clinical research
Cancer Registry	Human Resources	Cancer Prevention
Chief Medical Executive	Information Technology	Epidemiology
Critical Care	Information Management	Health Disparities
Ethics	Legal Services	
Gynecologic Oncology	Patient Education	
Infectious Disease	Patient Experience	
Medical Oncology	Patient Safety	
Occupational and Physical Therapy		
Psychiatry		
Radiation Oncology		
Social Work		
Surgical Oncology		

Participant comments on the most valuable aspects of the retreat emphasized the small-group discussions including having members of administrative leadership as participants. Attendees appreciated having time to work together to identify goals, discuss problems, and strategize improvements. As evidenced by comments from attendees, the mixed composition of the groups did not go unnoticed:

I liked the authenticity at all levels and the willingness to acknowledge the deficits we as an institution hold.

I liked hearing from SGM employees and their personal experiences here.

The retreat made me want more tools for building and maintaining a safe environment for SGM patients and staff.

Feedback from retreat evaluations indicated a high level of participant satisfaction. Attendees provided positive ratings for the organization and content of the retreat, and most respondents strongly agreed they would recommend the retreat to others (*M* = 4.65 out of 5, *n* = 20; Table 2). Attendees felt comfortable sharing thoughts, feelings, and/or lived experiences with other attendees (*M* = 4.65 out of 5, *n* = 20) and indicated they benefitted from the exchange of ideas during the small-group discussion (*M* = 4.6 out of 5, *n* = 20). Many respondents indicated that the retreat facilitated new connections with practitioners and researchers focused on SGM populations (*M* = 4.25 out of 5, *n* = 20). Several open-ended comments highlighted the “diversity of participants” and “passionate” speakers as valued aspects of the retreat.

**Table 2:** End-of-retreat evaluation

Question	Mean^a^	SD
The retreat was well-organized	4.75	0.43
The material covered during the retreat was appropriate for me	4.55	0.67
I would like to see the retreat remain in a hybrid format	4.25	0.94
I was able to connect with more SGM cancer practitioners/researchers than I knew before the retreat	4.25	0.89
I felt comfortable sharing my thoughts, feelings, and/or lived experiences with other attendees	4.65	0.73
I benefited from the exchange of ideas during the small group discussions	4.60	0.66
I remained engaged with the speakers/facilitators throughout the retreat	4.50	0.81
I left the retreat with more knowledge regarding SOGI data collection efforts at MD Anderson than I came with	4.35	0.65
Overall, I would recommend the retreat to others	4.65	0.48

^a^Scale: 1 = strongly disagree, 2 = disagree, 3 = neutral, 4 = agree, 5 = strongly agree

### Achievement of Retreat Objectives

Multiple sources of data indicated that the retreat objectives were generally met including identifying SMART initiatives that support SGM patient care and research.

- Retreat goals (2022):

1. Development of a Quality Improvement (QI) pilot to standardize SOGI data collection and staff training. Involved departments: Radiation Oncology, Nursing, Epidemiology, Ethics, Information Technology. Time frame: 12 months
2. Development of educational materials for SGM patients on sexual health-related toxicities of cancer care. Involved departments: Radiation Oncology, Surgical Oncology, Medical Oncology, Patient Experience, Patient Education. Time frame: 12–24 months

- Retreat goals (2023):

1. Developing biomedical and epidemiological research programs to incorporate SOGI data. Involved departments: Medical Oncology, Radiation Oncology, Epidemiology, Ethics, Clinical Research. Time frame: 12–24 months.

### Opportunities for Improvement

Approximately 50% of respondents shared suggestions for enhancing the retreat. In response, retreat organizers allocated more time for small-group sessions and problem-solving discussions while reducing the time for large-group activities and lectures. Attendees expressed a desire to hear more shared experiences of care at the institution of SGM patients and caregivers themselves, as well as updates on institutional progress.

I would like to see a potential roadmap, stories from patients about positive experiences, benchmarks and best practices from other organizations including Texas State Universities.

I would like to hear more patient stories of how we can be more inclusive.

I would like to see progress made from today’s discussions.

## Discussion

Organized by a diverse multidisciplinary team from departments across the institution, two half-day institutional retreats were successfully organized at a state university-affiliated cancer center aimed at enhancing healthcare, education, and research programs for SGM people with cancer. Attended by individuals from many departments holding a variety of roles, the retreats fostered safe discussion of and institutional reflection on current practices, attitudes, and opportunities for growth both across the institution and within departments and clinics. They also served as tangible representation of the diverse individuals from across the institution interested in the topic who were invited, without imposing institutional hierarchical structures, to contribute ideas for improvement. Indeed, the development of a QI pilot to collect SOGI data from new patients and design and deliver staff training in the Division of Radiation Oncology was directly attributable to the retreat. These efforts were pivotal for advancing efforts toward standardizing institution-wide practices and ensuring respectful and professional behavior provided by staff. As cancer centers strive to champion the needs of marginalized communities, this retreat framework offers an adaptable blueprint for healthcare organizations to catalyze institution-wide reflection and action on grass-roots initiatives, particularly on sensitive topics that may not be frequently discussed or prioritized by organizations.

## Data Availability

The authors declare that data supporting the findings of this study are available within the article. Additional information can be requested from the corresponding author (B.R.S.).
